# Non-Directional Property of Human–Body Communication Channel for Implantable Device Application

**DOI:** 10.3390/s23156754

**Published:** 2023-07-28

**Authors:** Jaehyo Jung, Daegil Choi, Da Eun Kim, Meina Li

**Affiliations:** 1 AI Healthcare Research Center, Department of IT Fusion Technology, Chosun University, Gwangju 61452, Republic of Korea; jh.jung@chosun.ac.kr (J.J.); daegilchoi0123@gmail.com (D.C.); k.daeun128@gmail.com (D.E.K.); 2 Department of Instrument Science and Technology, Jilin University, Changchun 130061, China; 3 Yibin Research Institute of Jilin University, Yibin 644000, China

**Keywords:** human–body communication, implantable device, channel measurement, impulse response

## Abstract

In this paper, we present the properties of a communication channel used for implantable devices. The human–body communication (HBC) channel was proposed for data communication in implantable devices. The impulse response was measured using a channel-mimicking model, which mimics electrical losses caused by human body tissues. Furthermore, we compared two types of channel-mimicking models to evaluate their applicability depending on the measurement environment. The resultant impulse responses of the HBC channel showed that HBC does not cause severe changes in the channel properties even when the implantable device is rotated.

## 1. Introduction

Implantable devices are essential for improving the quality of healthcare services. These devices are equipped with built-in sensors to monitor a wide range of biosignals, such as blood pressure, glucose levels, brainwaves, and neural signals [1,2]. Implantable devices require a communication channel to transmit data collected inside the human body. Due to the advancement in implantable technology and the increasing number of devices that can be implanted inside the human body, big data are being collected using these devices. Hence, a wider bandwidth is required for communication channels to transmit collected data from implantable devices at a high data transfer rate. Therefore, human–body communication (HBC) is an optimal transmission method for implantable devices. In HBC, the human body is used as a transmission medium because of its conductive properties [3,4,5].

HBC provides a wide bandwidth (up to 100 MHz) and utilizes low frequencies (less than 100 MHz). The conductivity of these tissues inside the human body decreases as the frequency increases [6]. Therefore, HBC has a smaller path loss for an implantable application than radio frequency transmission, such as the 2.4 GHz industrial-scientific-medical transmission. Prior to designing an implantable device, a channel should be investigated in terms of signal loss because it determines the required level of sensitivity at a signal receiver.

In previous studies, a channel-mimicking model used saline water that has almost the same salt concentration as that of the human body [3,4]. Several studies have used minced pork as a channel-mimicking alternative to saline water [7,8]. Recently, the channel properties of HBC have been presented in [9,10]; however, the properties were presented at a low-frequency band of 100 kHz, which is applicable to the body surface alone and not to an implantable device.

This study presents the HBC channel loss that was measured when using minced pork but over high frequencies up to 100 MH. An implantable device such as a capsule endoscope can freely rotate while passing through internal organs, thereby resulting in various signal losses in the channel. Thus, the experimental results for the HBC channel properties during device rotation are presented. Additionally, this study presents an experimental comparison between the channel-mimicking materials, such as saline water and minced pork, which are common alternatives for real tissues. This comparison is useful because either material can be used for experiments with implantable devices if they are compatible.

## 2. Materials and Methods

In our study, a capsule endoscope was considered as an application of HBC. For a channel measurement, a capsule device transmitting a channel measurement signal was fabricated, in which the device size was similar to that of a real capsule-type endoscope. After inserting the fabricated capsule into a channel-mimicking model, a receiving signal was measured outside the model, and signal loss was calculated using the transmitting and receiving signals.

**Figure 1:**
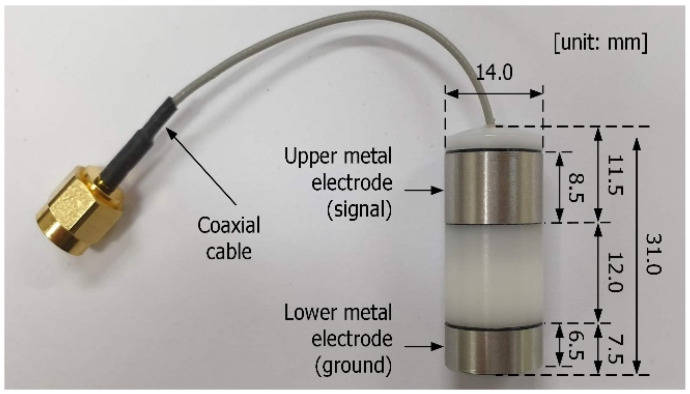
Structure of capsule device for HBC channel response measurement.

Figure 1 shows the capsule device used for the HBC channel response measurement. The cylindrical body was fabricated from polyacetal material with a dielectric constant of 3.7. Polyacetal was selected for capsule fabrication due to its excellent formability, even for small structures. HBC requires an electrode to transmit a signal through a medium such as the human body [3,4,5]. Based on the electrode’s structure, HBC supports two types of data transmission: capacitive and galvanic coupling [11]. As shown in Figure 1, the capsule used in this study consists of the signal and ground electrodes to facilitate galvanic coupling. Transmission by galvanic coupling is desirable for an implantable device because the signal and ground electrodes are used simultaneously, which results in a stable transmission channel between the implantable device and the receiver outside the human body.

As shown in Figure 1, the cylindrical body is sealed with two covers at the top and bottom. A small hole is drilled into one cover to insert a microcoaxial cable into the capsule. In coaxial cables, the signal and ground lines are connected to the signal and ground electrodes, respectively. This coaxial cable is used to drive the electrodes with a pulse signal that is transmitted through a channel-mimicking material. Small holes penetrating the cylindrical body are present beneath each electrode through which each line of the coaxial cable is connected to the electrodes.

**Figure 2:**
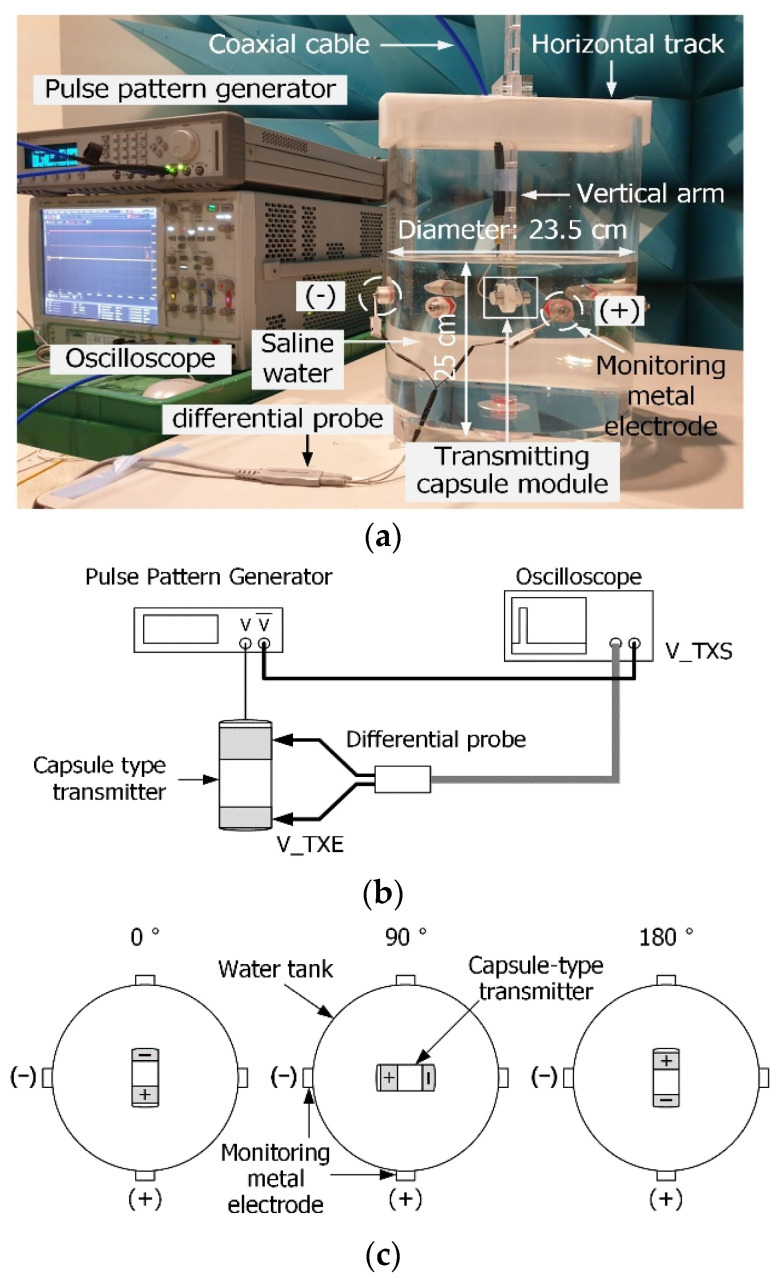
Measurement setup for HBC channel measurement. (**a**) Configuration of measurement equipment and channel-mimicking material, (**b**) Schematics of transmitted signal measurement using a differential probe, and (**c**) Capsule rotation.

Figure 2 shows the setup used for the HBC channel measurements. As shown in Figure 2a, the capsule is positioned at the center of the water tank, which contains saline water to mimic the HBC channel of the human body. The water tank is equipped with a vertical arm to hold the capsule device inside the channel-mimicking material and a horizontal track on the top. The vertical arm can move along the horizontal track. The vertical arm and horizontal track can adjust the position of the capsule device inside the channel-mimicking material.

The pulse-pattern generator generates a pulse signal that is transmitted to the capsule device through the coaxial cable. After driving the two electrodes on the capsule surface, the pulse signal is transmitted through the channel-mimicking material. Additional electrodes are placed on the tank surface to receive the transmitted pulse signals. The electrodes are connected to a differential probe, as shown in Figure 2a. The received signal is measured using an oscilloscope. The distance between the capsule device and the inner wall of the water tank was 11.5 cm. The water tank dimensions and the location of the metal electrodes were set to avoid excessive signal loss between the capsule and electrodes, enabling the measurement of the received signal at the electrodes.

Figure 2b shows the schematics of the transmitted pulse signal measurement using the differential probe. Prior to the signal measurement, the transmitted pulse signal was measured at the electrodes of the capsule device. The transmitted signal was used to calculate the impulse response after receiving the measured signal. The transmitted signals were measured at the electrodes for the enhanced accuracy of the measurement. As a result, the impulse response could be calculated more accurately.

Figure 2c shows the configuration of the capsule rotation. While measuring the received pulse signal, the capsule device was rotated horizontally about a fixed vertical axis. The received signals were measured at 0°, 90°, and 180° angles. Capsule rotation enabled the measurement of the variations in the HBC channel, similar to when an implantable device is randomly rotated inside the human body. Rotations of 90° and 180° yielded the worst results in the channel because the electrodes were aligned in perpendicular and inverse directions, respectively, after rotation.

## 3. Results

**Figure 3:**
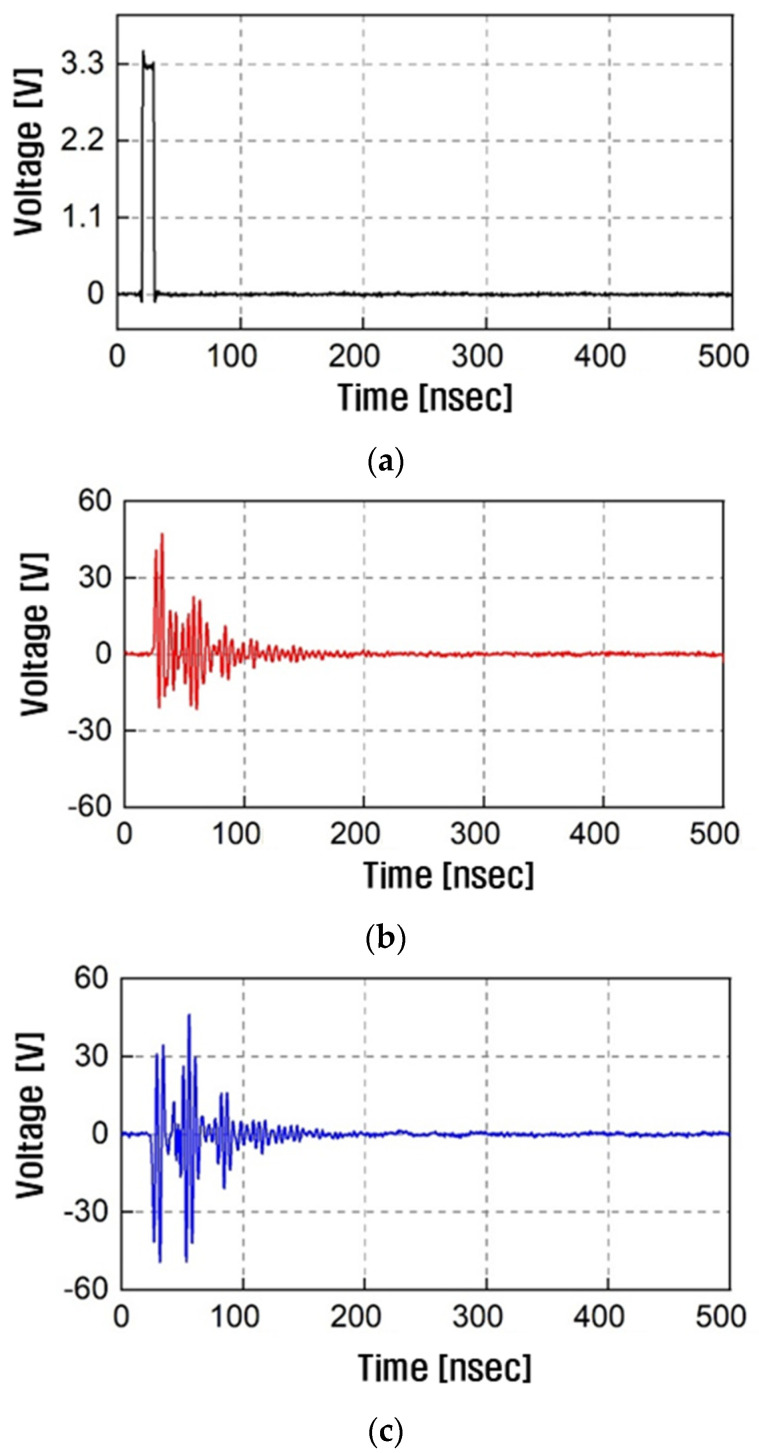
Measurement results for HBC channel: transmitted (**a**) and received pulse signal when the rotation angles were (**b**) 0° and (**c**) 180°.

Figure 3 shows the transmitted and received signals. For the transmitted pulse signal shown in Figure 3a, the pulse width and duty cycle were 10 ns and 0.1%, respectively, which is sufficient for measuring the HBC channel over 100 MHz. Then, the received signal was allowed to decay to zero amplitude. The received signal must be measured with respect to the transmitted pulse signal to calculate the phase response of the HBC channel. The oscilloscope shown in Figure 2b was triggered during the received-signal measurement. Figure 3b,c show the measured received signals when the capsule was rotated by 0°and 180°, respectively. The received signal varied with the capsule rotation due to the change in the transmission distance between the capsule and receiving electrodes on the surface of the water tank.

After measuring the transmitted and received signals, each signal was transformed from the time domain to the frequency domain using Fast Fourier transform (FFT). For FFT, the sampling rate and windowing width were 20 G samples/s and 200 ns, respectively.

**Figure 4:**
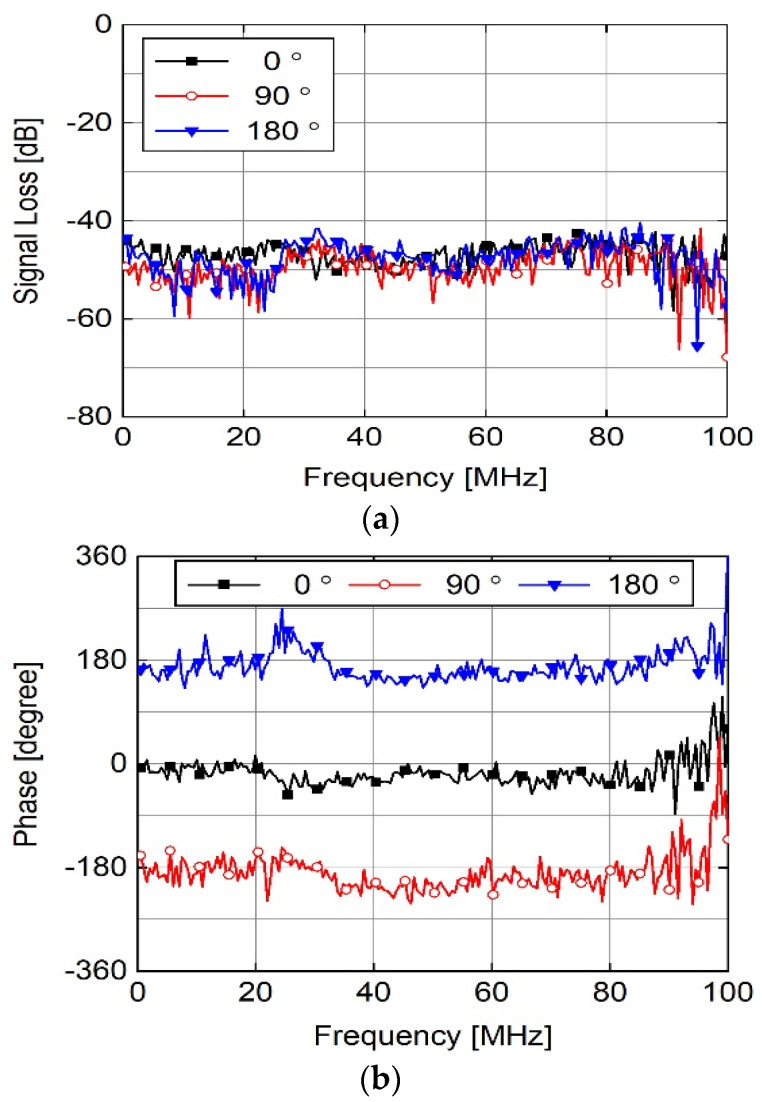
Measured signal loss of HBC channel. (**a**) Magnitude and (**b**) Phase response.

The transformed signals were subtracted to obtain the magnitude and phase responses of the signal loss in the HBC channel. Figure 4 shows the magnitude and phase responses. As shown in Figure 4a, no significant variation was observed in the magnitude of the signal loss for capsule rotation. The variation was less than 4 dB for all frequencies up to 100 MHz, while the mean absolute errors were 3.80 dB and 3.10 dB for 90° and 180°, respectively, relative to 0°.

This is consistent with the received-signal measurement results shown in Figure 3b,c. As shown in Figure 4a, the signal loss was approximately 52 dB at 21 MHz, which is the center frequency defined by the IEEE 802.15.6 HBC standard [12]. However, the phase response changed according to the capsule rotation. The variation in the phase response was caused by the change in the relative polarity of the electrodes; the capsule electrode at the shortest distance from one of the water tank electrodes exhibited a polarity change according to the capsule rotation. The transmission distance did not change significantly, whereas the relative polarities of the capsule electrodes did. Therefore, the magnitude was almost constant; however, the phase response was varied.

**Figure 5:**
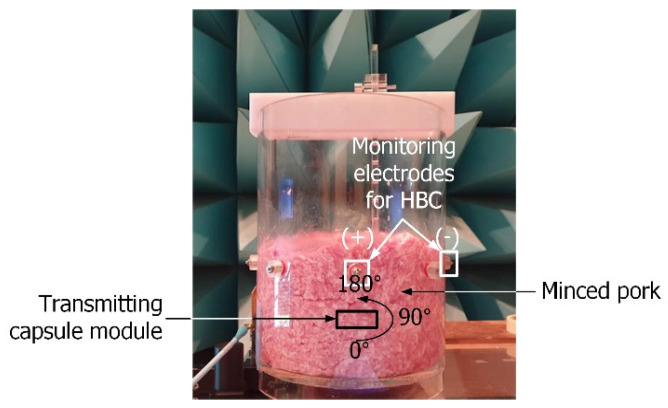
Channel-mimicking model involving minced pork.

For the comparison of the two channel-mimicking materials, the magnitude of the response of the HBC channel was measured in a similar manner after changing the medium from saline water to minced pork. Figure 5 shows the channel-mimicking material containing minced pork. The same water tank was filled with minced pork instead of saline water. The capsule device was positioned inside the channel-mimicking material, and the received signal was measured at the electrodes on the surface of the water tank.

**Figure 6:**
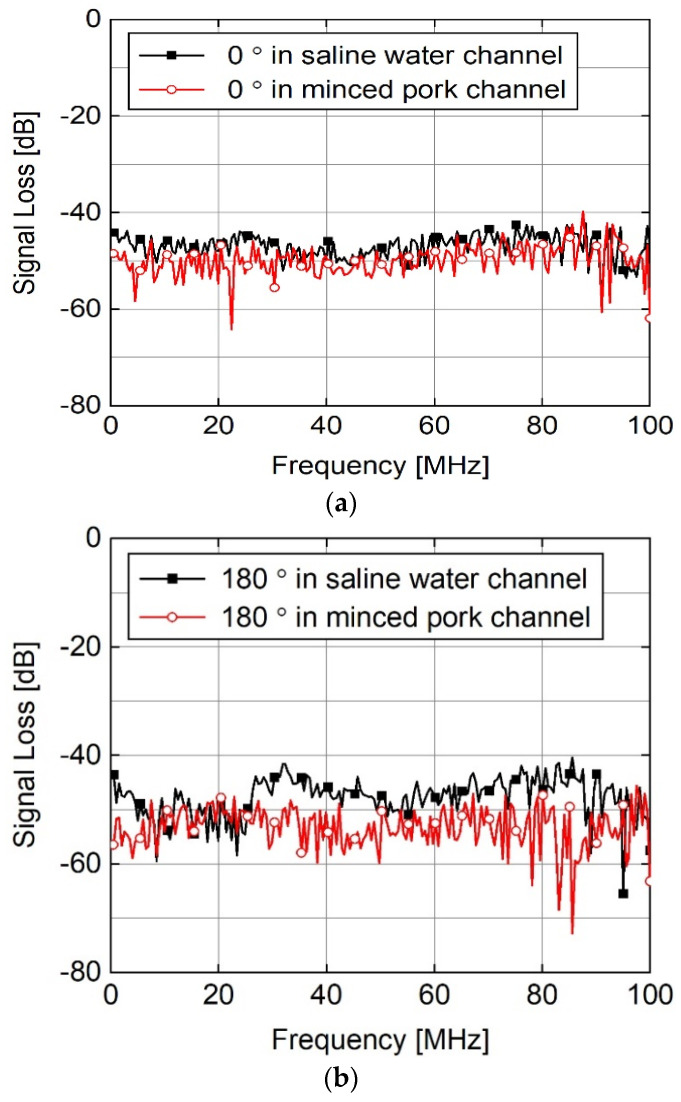
Comparison of magnitude response according to the channel-mimicking material when rotation angles of the capsule were (**a**) 0° and (**b**) 180°.

Figure 6 shows a comparison of the magnitude responses when the channel-mimicking material was changed from saline water to minced pork. The results in Figure 6 indicate that the material change increased the magnitude of signal loss. Minced pork is a realistic material as it represents real human body tissues with higher accuracy than saline water.

Table 1 lists the average signal losses in the in-body to on-body channels for the two types of channel materials. The mean signal losses were calculated by averaging the measured signal losses at all measurement frequencies up to 100 MHz. However, the average change in the signal loss was less than 3.32 dB, thereby indicating that saline water could replace minced pork in mimicking the tissues of the human body for HBC channel measurements.

Saline water is desirable for HBC channel measurements as it can be prepared more easily than minced pork. This provided additional flexibility for capsule placement because it was possible to move the capsule device more freely inside the material. The comparison results shown in Figure 6 are useful for selecting the channel-mimicking material between saline water and minced pork, depending on the measurement environment.

The impulse response of an HBC channel can be obtained from the signal loss, which comprises the magnitude and phase responses. The signal loss was transformed into the time domain by applying an inverse FFT in the frequency domain.

**Figure 7:**
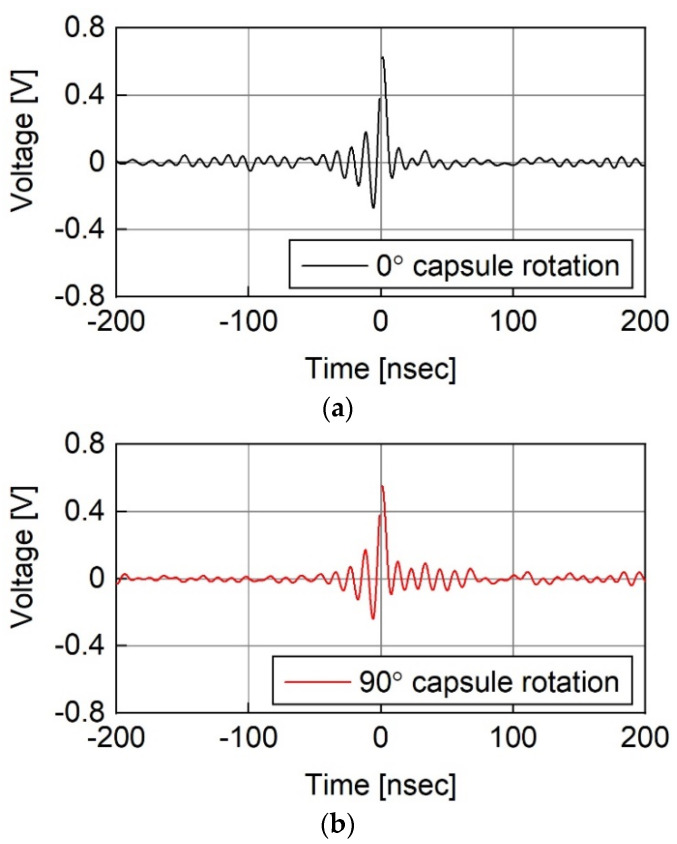

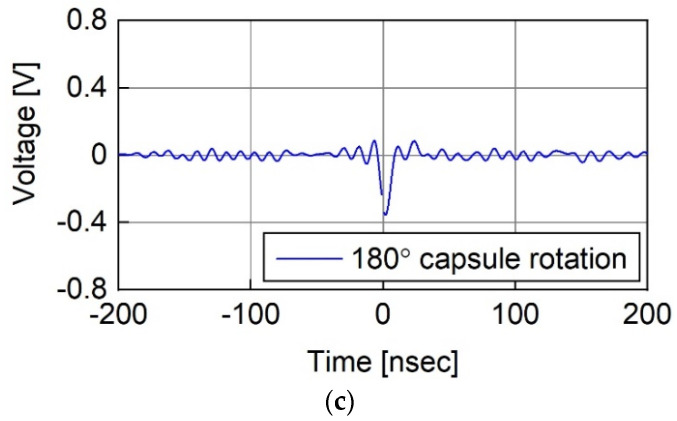
Impulse response of HBC channel measured in minced pork when capsule rotation angles were (**a**) 0°, (**b**) 90°, and (**c**) 180°.

Figure 7 shows the impulse responses of the HBC channel obtained from the measurements with minced pork as a medium. As shown in Figure 7, the impulse response changes are in relation to the capsule rotation. However, the change is not significant because all responses have an absolute peak value of approximately 0.4 V and a pulse duration of approximately 10 ns. The constant response is consistent with the signal loss measurement results shown in Figure 4.

## 4. Conclusions

In this study, we compared the channel-mimicking materials used in HBC channel response measurements for data communication in implantable devices. The measurement results showed that the two channel-mimicking materials had similar magnitudes of signal losses but different phase responses based on the model used. The measured signal loss was approximately 52 dB, which is the center frequency defined by the IEEE 802.15.6 HBC standard. This indicated that compatibility existed between the channel-mimicking materials, and either material could be used depending on the experimental environment. The difference in the signal loss was minimal even when the device was rotated; therefore, the HBC channel could be beneficial because the movement of an implantable device within the internal organs would not cause significant variations in the magnitude of signal losses.

## Figures and Tables

**Table 1:** Average signal loss for the in-body to on-body channel.

Channel Material	0°	180°
Saline water	−46.62 dB	−47.08 dB
Minced pork	−46.62 dB	−52.56 dB

## Data Availability

The study does not report any data.

## References

[1] Nelson B.D., Karipott S.S., Wang Y., Ong K.G. (2020). Wireless technologies for implantable devices. Sensors.

[2] Bazaka K., Jacob M.V. (2012). Implantable devices: Issues and challenges. Electronics.

[3] Hyoung C.H., Hwang J.H., Kang S.W., Park S.O., Kim Y.T. (2015). A feasibility study on the adoption of human body communication for medical service. IEEE Trans. Circuits Syst. II Express Briefs.

[4] Roopali P., Kumar R. (2018). Technological aspects of WBANs for health monitoring: A comprehensive review. Wirel. Netw..

[5] Intromedic ‘Microcam’. https://www.intromedic.com:549/eng/item/item_010100_view.asp?search_kind=&gotopage=1&no=3.

[6] Dielectric Properties of Body Tissue. http://niremf.ifac.cnr.it/tissprop/.

[7] Liu K., Liu R., Cui W., Zhang K., Wang M., Fan C., Zheng H., Li E. (2021). Design of Conformal Spiral Dual-Band Antenna for Wireless Capsule System. IEEE Access.

[8] Yan L., Guo Y.-X., Xiao S. (2017). Orientation insensitive antenna with polarization diversity for wireless capsule endoscope system. IEEE Trans. Antennas Propag..

[9] Zhang S., Pun S.H., Mak P.U., Qin Y.P., Liu Y.H., Gao Y.M., Vai M.I. (2019). Experimental Verifications of Low Frequency Path Gain (PG) Channel Modeling for Implantable Medical Device (IMD). IEEE Access.

[10] Maity S., He M., Nath M., Das D., Chatterjee B., Sen S. (2019). Bio-Physical Modeling, Characterization, and Optimization of Electro-Quasistatic Human Body Communication. IEEE Trans. Biomed. Eng..

[11] Callejon M.A., Naranjo-Hernandez D., Reina-Tosina J., Roa L.M. (2013). A comprehensive study into intrabody communication measurements. IEEE Trans. Instrum. Meas..

[12] (2012). IEEE Standard for Local and Metropolitan Area Networks—Part 15.6: Wireless Body Area Networks. https://ieeexplore.ieee.org/stamp/stamp.jsp?arnumber=6161600.

